# Editorial

**Published:** 1856-06

**Authors:** 


### Editorial.

VALEDICTORY.

In closing this, the Ninth Volume of the "Dental Register of the West," we respectfully say to our readers, Farewell. We have been sending you now, for nine long years, our quarterly greetings, and yet it appears only as so many months. Time flies, how rapidly! Let us take a retrospect and see what has been accomplished. Our first salutation was a small pamphlet of forty-eight pages, and was sent forth with much fear and solicitude,—fear that the profession was not large enough in numbers to sustain even such a work, and solicitude that original articles could not be procured to make it interesting and instructive.

Our profession was known to be composed of hard working, energetic, persevering men, many of whom would far rather *work* out a difficult problem than put the results on paper—men who were hard at work knocking off an incalculable amount of rubbish; which they felt, soon would reveal a science which would take its place side by side with the noblest of the earth—one of which its parent (Medicine) might well be proud.

Many of these were known to be more disposed to the practical detail of the profession, than its literary embellishment—men who felt far more solicitude how they might embellish the mouth with precious gems of utility and beauty, than to enrich with the most precious *gems* by theoretical discussion its literature. Under these circumstances, it is with feelings of no little gratification we take a retrospect of the past nine years and compare the enlargement of the Register to the advance which has been made in dental science and its literature.

The Dental Register has not more than kept pace with the progress of the profession, and now it is not only in size more than twice as large as when first started but in every other respect its ratio of improvement would bear a similar comparison. In claiming this for the work we have been superintending, we pretend not to claim any special merit to *ourself*, but freely and cheerfully claim it for the profession, so many of whom have, by their contributions and otherwise, aided us in our labors.

We claim, however, through the pages of the Register, a very pleasant and profitable acquaintance with a great many of the profession, with whom we nominally part with regret; and yet we hope for many long years to see their names in its pages, and if health permit, we hope *thus* still to claim a continued acquaintance.

We have ever felt it a duty we owe to the profession to contribute our mite for its advancement. It is one of the important objects of our periodicals to gather up the large and little improvements, the odds and ends of the profession, and preserve them for reference and ultimate compilation in a more permanent form.

It will be observed that the little improvements exceed very much the larger ones. In the aggregate they swell out and make a far larger show. They are indeed the gradual openings of the flower. The large improvements are truly nothing more than the last unfolding of the flower. Our periodicals collect and preserve these little advancements, stimulate the ambitious to improve upon them, and thus are constantly developing, by their silent influence, an elevating and progressive spirit. It is owing to this fact that they are invaluable to the dental practitioner. They are far more valuable than many suppose. The fact is, there is not a man, however high his position, who is not deeply indebted to our periodicals. If he is not he must be ten years behind the times. It is true he may never read them but he has *heard* them, and if he has made any advance at all they have aided him.

Taking the view we here present, and it is a true one, we must feel it our duty to sustain such publications. We do not see how any man with a spark of professional pride can refuse to give his support to such a cause. We do not say that every man should take all such publications, this would often be too much of a tax; but every man should take one, and 'many should take more. As we now retire from the labor, care and anxiety of such a work, we may be allowed to speak freely on this subject, and *en passent* we express our—we will say *pity*, for those who very graciously receive such publications for perhaps years, yet when a little bill is sent they flare up in a great passion and refuse to pay, say they never read them and that they never subscribed. The *law* says if they take them from the office they are subscribers, and the law is right. The publisher sends you his work; you know his terms and you receive it from him. He has done his duty and you should do yours. Courtesy required you to send it back if you did not intend to pay for it.

It should be recollected by the profession that our periodicals cannot have a large circulation, and it is owing to this fact that they cannot be afforded at what might with many be considered a cheap rate, yet if we come to compare our dental periodicals with the medical, they are perhaps fully as cheap. The profession have it in their power, however, to make such publications even cheaper than they are. Sustain them well and they will be increased, in not only size but real value. They should be sustained, not only by prompt pay, but by regular and interesting articles for publication. There are now enough who can write, and write well, to sustain three or four such works with credit to the profession.

We know of nothing, so far as our experience goes, which would give those who edit and have published our dental periodicals more real pleasure than to have every man who does not want and will not pay for such works, to carefully remail them to the address of the paper with their names written plainly on the work or envelope, and the word refused also written thereon. This is perhaps the postmaster's duty, yet they often neglect this, and even whern it is attended to it is often done so that no man living can tell from whence it comes. We have, for instance, had the Register returned, and perhaps the word *refused* written on it, yet the name to whom it was sent, nor the post-office, was not on it.

We have thus spoken freely of some of the vexations of a journalist, and have done it more especially for the benefit of those we now leave to assume the duties we have tried in a feeble manner to perform. We feel that it is a great work, one requiring much energy, perseverance, and, we may add, talent; and we are happy to assure our readers that the gentlemen into whose hands we place the Register possess in an eminent degree these requisites.

Drs. Taft & Watt are both favorably known to the profession; they enter upon the editorial duties of the Register, with the determination to leave no stone unturned by which it may be made just such a work as the fast increasing growth of the profession in this great valley demand. We cheerfully commend them to the profession and bespeak for them all that assistance from the liberal of the profession which so important an enterprise should receive.

We feel that we cannot, in justice, close these remarks without expressing our thanks to the numerous friends of the Register, who have ever from the first taken a deep interest in its success. These friends have, we are glad to say, increased in numbers yearly, and now furnish a somewhat reliable support, insuring its countinued prosperity. We would also remember the fostering care of the Mississippi Valley Association, under whose auspices the work was started and whose fostering care sustained it in part for the first five years.

To the editorial fraternity we express our thanks for the kind courtesy extended. We have ever felt an interest in every effort by the profession to disseminate useful knowledge, and have aimed in all cases to give due credit to other works of a like character, and we believe in only one or two instances has this been omitted, and then it was through the oversight of the printer.

We are glad to say to our readers that it is not from ill health, old age nor a want ot disposition which induces us to relinquish a work which we have found in most respects very pleasant, but it is from the fact that our office duties, and those connected with the college, so completely tax our time and energies that we have not that time to devote to the Register which its increasing circulation and size demands. We wish, if possible, more time to devote to that department of dental science it is now our duty to teach (we mean Institutes of Dental Science). We feel that there is ample room here for much study and research, and if our pen will still *scribble* we wish it guided in this channel. JAMES TAYLOR.

THE DENTAL REGISTER.

The present number closes the ninth volume, and, as it may be seen, our connection with the same. We would here say, that those of our subscribers who are deficient in numbers to make up full sets, for which they have paid, that they can be supplied with any numbers that may be missing, except the October and January numbers of volume eight. If any of the profession have extra numbers of these they would confer a great favor by sending them to the address of the Dental Register, Cincinnati, Ohio.

We would also say to the profession, that a few sets of the Register, from the first are still for sale, and will be furnished at $1 50 per volume, for the first eight volumes. Drs. Taft & Watt will furnish subscribers with the Register from the beginning of volume nine, if desired ; also, an order through them will secure any of the back numbers wanted, the same as by direct application to ourself.

---

SUBSCRIBERS.

Those who are still indebted for the Register are notified that their accounts are turned over with the Register to Drs. Taft & Watt. Their receipt will therefore be perfectly valid in all settlements.

Mr. James, whose agency was noticed in the last number, will still attend to the collecting of bills. His or his agents receipts will always be considered good.

We hope that all who are in arrears will make a special effort to square their accounts. If any prefer sending the amount of their bills direct to us, we will send receipts and attend to them with pleasure.

---

ORAM & ARMSTRONG'S TEETH.

The teeth of this establishment have been gradually gaining more and more the confidence of the profession. We are glad to say that within the last year there has been a decided improvement in their strength and their ability to resist the action of heat.

These improvements have been made without in the least affecting their beauty. These gentlemen certainly deserve a great deal of credit for the energy with which they are trying to meet the demands of the profession.

With two such establishments as we now have—Messrs. Oram & Armstrong, and Messrs. Jones, White & McCurdy—the profession need desire but little more. The competition between these two establishments bids fair to be of service to both, and equally so to the profession.

Oram & Armstrong's teeth are for sale in this city by S. Palmer, Fourth street.

---

THE AMERICAN DENTAL CONVENTION.

We hope the readers of the Register will bear in mind that the "American Dental Convention" will meet in New York on the Sixth (6th) of August

This is now by far the most important Dental Association in existence. Gotten up on an enlarged and liberal basis, it is calculated to embracee the whole profession. The proceedings and discussions must be witnessed and heard to be fully appreciated. The fullest report that the journals can give, is at best but a meagre outline of the progressive truths eliminated by the discussions We therefore hope our readers of the South and West will improve this occasion to treat themselves to a visit east ; and we feel assured that those who do so, will return to their patrons with refreshed energies, renewed vigor, and enlarged ideas of professional truths. Let us endeavor to make this truly a *national* association. How many will go ?

---

### AMERICAN SOCIETY OF DENTAL SURGEONS.

This *parent* society stands adjourned to meet in New York on the 5th of August. We hope and trust that the members will endeavor to be *all* on hand. The business postponed from last year is important. We hope the attendance will fully demonstrate the fact that whatever reasons the society may have for discussing the propriety of its dissolution, a want of vitality is not one of them. And if the society should find that its office is fulfilled, that its work is done, aud that other associations have arisen under its parental care, better adapted to the present wants of the profession, let its departure be regarded as a resignation, not as a dissolution. And should it retire from active life, still it can never die. It will live in the literature of the profession. It must live, because the world is the better for its having lived.

Should it. however, resolve to continue in active duty, may its future triumphs be equal to its past success.

---

*The Mutual Responsibilities of Physicians and the Community. An Address to the Graduating Class of the Medical College of the University of Michigan.* By Henry P. Tappan, D.D., L.L D., Chancellor of the University of Michigan.

We have long regarded this subject as worn thread-bare ; but, as in the progressive spirit of the age old clothes are renovated, and shine like new, so in reading this address, we forget the staleness of the subject while admiring the newness of its dress. The subject has been mostly discussed by the physicians—this is by one of " the community," and the view is therefore presented in a new aspect

---

*Proceedings of the Convention and of the Medical Society of the State of California, held in Sacramento, March,* 1856.

A *State* Medical society on the Shores of the Pacific ! Sixteen counties represented, with a delegation of twenty-eight members from a single county! Who says the medical profession is destitute of energy ? The Convention organized and proceeded to the formation of a State Society, by adopting a constitution, by-laws and a code of ethics, the latter being that of the "American Medical Association."

Dr. B F. Keene, of El Dorado, is President, and Dr. Thomas M. Logan, of Sacramento, Corresponding Secretary.

---

*Transactions of the South Carolina Medical Association, at the extra meeting in Greenwood, July* 18*th*, 1855, *and at the Annual Meeting in Charleston, February* 6*th*, 1856.

This is an interesting document of over seventy pages, containing much that is important in the shape of essays, reports of cases, etc. Our space will not permit even an outline of its contents. We trust those interested will secure the document and examine for themselves.

The President of the Association is Dr. Horlbeck ; and Dr. F. M. Robertson, is Corresponding Secretary. Printed by Walker & Evans, Charlestou, S. C.

*The Peninsular Journal of Medicine and the Collateral Sciences ; Edited by* Zina Pitcher, M.D., Emeritus Professor of Obstetrics, &c., in the University of Michigan. A. B. Palmer, M.D., Professor of Materia Medica, &c., in the University of Michigan, and Wm. Brodie, M. D., and Edmund P Christian, A.M., M.D., Assistant Editors.

The present [June] number completes the third volume of this Journal. We hope the editors will enter on the fonrth volume with the encouragement their efforts merit. With such an array of talent in its editorial corps, the Journal can scarcely fail to prove highly instructive and interesting.

Printed for the Proprietors by W. F. Story, Detroit, Michigan.

---

### RECEIPTS FOR THE REGISTER.

Dr. J. F. Warren, Terre Haute, Ia....... $3.00
Dr. H. R. Smith, " " "...... 3.00
Dr. C. W. Spalding, St. Louis,.......... 3.00
Dr. H. Barron, " .......... 6.00
Dr. A. Blake, " .......... 7.00
Dr. J. Paine, " .......... 4.00
Dr. H. J. B. McKellops," .......... 5.00
Dr. I. Forbes, " .......... 9.00
Dr. R. Somerby, Louisville, Ky......... 3.00
Dr. H. M. Lewis, Quincy, Ill............ 3.00
Dr. A. Hollowbush, " .......... 14.00
Dr. G. S. Miles, Jerseyville, Ill.......... 3.00
Dr. P. G. C. Hunt, Indianapolis, Ia..... 9.00
Dr. E. Townsend, Philadelphia, Pa...... 3.00
Dr. H. G. Hall, Muscatine, Iowa, ....... 2.00
Dr. Daniel Dougherty, Beardstown, Ky.,. 3.00
Dr. A. L. Brown, London, Ohio,........ 3.00
Dr. J. B. Newbro, Cincinnati, Ohio,..... 1.00
Dr. J. H. Andrews, Yazoo, Miss......... 3.00
Dr. L. Levett, St. Louis, Mo............ 2.00
Dr. H. E. Peebles, Lexington, Mo....... 5.00
Dr. J. H. Blager, Mt. Pleasant, Iowa,... 3.00
Dr. W. H. Goddard, Louisville, Ky. .... 5.00
Dr. W. J. Wickfield, Sidney, Ohio,...... 5.00

